# Analysis of the community composition and diversity of endophytes in extremely spicy industrial chili peppers from Tibet using high-throughput sequencing

**DOI:** 10.3389/fmicb.2025.1630090

**Published:** 2025-09-01

**Authors:** Junze Zhang, Yebing Yin, Yanying Wang, Sicen Luo, Yu Li, Wenxiang Zhao, Pengxi Cao, Yixuan Liu, Hongmei Ma

**Affiliations:** Key Laboratory of Biodiversity and Environment on the Qinghai–Tibetan Plateau, Ministry of Education, School of Ecology and Environment, Xizang University, Lhasa, China

**Keywords:** industrial chili peppers, capsaicin, high-throughput sequencing, microbial community diversity, microbial community composition

## Abstract

Industrial chili peppers contain more than 100 times the capsaicin content of common chili peppers; these peppers are primarily used for industrial processing and capsaicin extraction. Chili peppers thrive in warm temperatures, require plenty of sunlight, and are drought-resistant; therefore, making the high-altitude climate of Tibet ideal for their cultivation. Endophytes are microorganisms that can inhabit healthy plants at various stages of their life cycle. Through long-term co-evolution, endophytes and host plants establish a mutually beneficial symbiotic relationship, which assists plants in secondary metabolite production. This study investigated the differences in endophyte community structure across various lines of industrial chili peppers. It also explored the relationship between capsaicinoids and endophyte community composition in high-altitude habitats of Tibet using high-throughput sequencing to obtain fundamental data on industrial chili pepper endophytes. The results showed that the diversity of endophyte communities was characterized by conservatism among groups and that the composition and community structure of endophyte communities were specific to different groups. Community composition analysis revealed that there were generally consistent dominant phyla of endophytic microorganisms in industrial chili peppers, although differences in their relative abundance percentage were observed. Bacterial community composition at the genus level was less affected by capsaicin concentration across different groups; however, the fungal community composition at the genus level was more responsive to capsaicinoid concentrations than that of bacteria. Bacterial communities from four different chili pepper varieties showed significant differences in the enrichment of genera. Fungi were differentially enriched in two groups: the td1 group with high capsaicin concentrations and the sylj group with low capsaicin concentrations. Among the four groups, endophytic bacteria exhibited the highest percentage of genes associated with unknown functions, while fungal trophic patterns had the most significant percentage of unknown trophic types. Overall, this study provides a valuable reference for the efficient cultivation and utilization of industrial chili peppers in Tibet.

## 1 Introduction

Industrial chili pepper is a new variety of chili from India, formerly known as “Spicy Multi No. 1.” It is an extremely spicy chili developed through extensive trial plantings and crossbreeding with local Chinese varieties. It has been widely popularized and planted in recent years. The capsaicin content of common edible chili peppers ranges from 0.01–0.5%, whereas the capsaicin content of the Chinese chili pepper hybrid (*Capsicum chinense* Jacquin) can reach from 14–18%, which makes it unsuitable for direct consumption and is mainly used for industrial processing. Therefore, it is called the industrial chili pepper (Yongxiang and Guangmei, 2020; Lei et al., 2024). Capsaicin and red pigments extracted from industrial chili peppers are mostly used in industry: capsaicin can be used for producing military tear gas and anti-corrosion agents for ships; the red pigment can be used in cosmetics and medicine (Xu et al., 2008). China is the largest producer of chili peppers worldwide, with an annual planting area of 600,000 hm^2^, accounting for 40% of the global chili pepper planting area (Song et al., 2023). As chili peppers are native to South America, they are temperature-loving, light-loving, and drought-resistant; therefore, strong light and moderate drought can significantly increase their capsaicin content, and the climate in Tibet is well-suited for chili pepper cultivation. Industrial chili pepper planting is simple, with a wide range of applications, and has a large market demand. It is especially suitable for planting in high-altitude areas where the yield of other crops is low, and can thus promote local economic development (Jianglong, 2019; Zhongcai, 2021).

Endophytes, which typically include bacteria and fungi, can live in healthy plants at some point in their life cycle (Rana et al., 2020). Endophytes are present in almost all plants and vary according to plant species, genotypes, and growth environments, making them a widely distributed group of microbial resources (Hardoim et al., 2015). During long-term co-evolution, endophytes and host plants have established a mutually beneficial symbiotic relationship which promotes host plant growth, assists the plant in secondary metabolite production, and increases its resistance to environmental stress (Kuldau and Bacon, 2008). Notably, plant endophytic fungi indirectly affect the production of host plant metabolites by altering their nutrient patterns at different stages of plant growth and development (Hou et al., 2022). Plant endophytic bacteria are involved in plant metabolite production mainly through metabolic functions such as substance synthesis (Tian and Zhang, 2017; Narayanan and Glick, 2022). This connection is vital between endophytic bacteria and plant physiology, ecological adaptation, and medicinal value. In addition, the abundance of secondary metabolites in the host plant shows a significant positive correlation with the abundance of dominant endophytic bacterial genera. For example, the metabolite levels of *Gentiana scabra* Bunge fluctuated with the structure of the endophytic bacterial community (Hou et al., 2022; Narayanan and Glick, 2022). Currently, due to high altitude, strong radiation, cold climate, and other extreme environments on the Tibetan Plateau, researchers have studied endophytes of plants such as *Leontopodium nanum* (Hook. f. & Thomson ex C. B. Clarke) Hand.-Mazz. (Liang, 2019), *Hordeum vulgare* var. *coeleste* Linnaeus (Liu et al., 2016), *Stipa purpurea* Griseb. (Bao and Li, 2016), and *Hippophae tibetana* Schltdl. (Zhang et al., 2018). These include both wild plants and cash crops; however, no relevant reports are available on the diversity of endophytes and the association between endophytes and capsaicinoids in industrial chili peppers on the Tibetan Plateau. Therefore, in this study, based on the high-altitude habitat of Tibet, we used industrial pepper as the research object and sequenced its endophytes using high-throughput sequencing technology to examine the differences in endophyte community structure among different lines of industrial pepper, as well as the relationship between capsaicinoids and the diversity and community composition of endophytes. To obtain basic data on the endophytes of industrial chili peppers, this study provides a theoretical foundation for the comprehensive utilization of microbial resources in Tibet, as well as data support for the efficient planting and cultivation of industrial chili peppers in the region.

## 2 Materials and methods

### 2.1 Regional overview and sample collection

This study was conducted in October 2024 at the Tibet Plateau Seed Industry Demonstration Base, Caina Township, Qushui County, Lhasa City, Tibet Autonomous Region, to collect the industrial chili peppers grown therein. Industrial chili pepper mother plants were cross-bred by Zhu Zhangsheng’s group from South China Agricultural University. For each type of industrial pepper, three healthy and mature plants were selected as biological replicates, and each sample was divided into roots, stems, and fruits (Dombrowski et al., 2017). A total of 48 valid plant samples were obtained: roots (16 copies), stems (16 copies), and fruits (16 copies). They were placed in sterile self-sealing bags and divided into four groups according to their capsaicinoid concentrations: high capsaicin concentration (td3), medium capsaicin concentration (td2), low capsaicin concentration (td1), and edible peppers (sylj). The samples were wrapped in sterile self-sealing bags and stored in a vehicle refrigerator (Mobicool, CF-50) at −20 °C. The samples were then returned to the laboratory for further analysis. At the laboratory, surface sterilization of plant tissues was performed by rinsing with sterile water for 30 s, followed by rinsing with 70% alcohol for 2 min, soaking in a 2.5% NaClO solution with 0.1% Tween80 for 5 min (Guo et al., 2022; Guo et al., 2023), then soaking in 70% sterile alcohol for another 30 s, and finally, washing three times with sterile water. Subsequently, the surface-sterilized samples and inter-root soil samples were stored in an ultra-low temperature refrigerator (Jiangsu Shenglan, DW86L-158) at −80 °C until further analysis.

### 2.2 Total DNA extraction, PCR amplification, and high-throughput sequencing of endophytes

Total microbial genomic DNA was extracted from industrial chili pepper samples using the E.Z.N.A.^®^ soil DNA Kit (Omega Bio-Tek, Norcross, GA, United States) following the manufacturer’s instructions. The DNA quality and concentration were determined using 1.0% agarose gel electrophoresis and a NanoDrop2000 spectrophotometer (Thermo Scientific, United States), and the samples were stored at −80 °C until further use. The hypervariable region V5–V7 of the bacterial 16S rRNA gene was amplified using the primer pair 799F (5′-AACMGGATTAGATACCCKG-3′) and 1193R (5′-ACGTCATCCCCACCTTCC-3′). The PCR amplification mixture (total volume 20 μL) comprised the following components: 5 × FastPfu Buffer 2 μL, 2.5 mM dNTPs 2 μL, 0.8 μL of each primer (concentration 5 μM), FastPfu Polymerase 0.2 μL, BSA 0.2 μL, template DNA 10 ng, and ddH_2_O to make up the remaining volume to 20 μL. All thermal cycling steps were as follows: 95 °C for 3 min; then 27 cycles of 95 °C for 30 s, 55 °C for 30 s, and 72 °C for 45 s, with a final extension of 10 min at 72 °C. Subsequently, a second round of amplification was carried out, consisting of 13 cycles under the same conditions as the previous round. Once the program finished, the samples were stored at 10 °C to ensure stability (Beckers et al., 2016).

High-throughput sequencing of the endophytic fungi of industrial chili peppers was performed using the ITS1 region for specific amplification with the primers ITS1F (5′-CTTGGTCATTTAGAGAGGAAGTAA-3′) and ITS2R (5′-GCTGCGTTCTTCATCGATGC-3′). The PCR amplification mix was composed of 10 × Buffer 2 μL, 2.5 mM dNTPs 2 μL, each primer (concentration 5 μM) 0.8 μL, rTaq Polymerase 0.2 μL, BSA 0.2 μL, template DNA 10 ng, and ddH_2_O to make up a total volume of 20 μL. The amplification process was as follows: 95 °C for 3 min; followed by 37 cycles of 95 °C for 30 s, 55 °C for 30 s, and 72 °C for 45 s, with a final extension at 72 °C for 10 min. Afterward, the sample was cooled to 10 °C to keep the product stable (Blaalid et al., 2013).

The purity and concentration of PCR products were subsequently assessed using a NanoDrop^™^ One (Thermo Fisher Scientific) ultra-micro spectrophotometer, and the DNA integrity was examined by 2% agarose gel electrophoresis. Sequencing was carried out on an Illumina MiSeq platform, and PCR amplification and sequencing were performed by Shanghai Majorbio Bio-Pharm Technology Co., Ltd. The raw sequencing data have been uploaded to NCBI under accession number PRJNA1259232.

### 2.3 Data analysis

Bioinformatics analysis was performed by Shanghai Majorbio Bio-Pharm Technology Co., Ltd. using the I-Sanger Cloud Platform.^1^

Raw sequencing data were spliced using Flash software (version 1.2.7), filtered using Trimmomatic software (version 0.33), and annotated based on Silva (Release 128, http://www.arb_silva.de) and Unite (Release 7.2, http://unite.ut.ee/index.php) databases. The operational taxonomic units (OTUs) were then classified using Usearch software (version 7.0) with 97% sequence similarity (Edgar, 2013).

Mothur software (version 1.30.1) was used to calculate the alpha diversity index and construct the rarefaction curve. R software (version 3.5.1) was used to analyze differences between groups using the t-test for the alpha diversity index.

Using Qiime software (version 1.17), the beta diversity distance matrix was calculated. The results of the ANOSIM intergroup difference test were combined to analyze the samples using non-metric multidimensional scaling (NMDS) (Wang et al., 2007).

Linear discriminant analysis effect size (LEfSe) for multilevel species difference was used to evaluate colonies that significantly influenced sample differences. Linear discriminant analysis (LDA) was used to screen colonies that were significantly different from each other. The degree to which environmental factors explained the different functions of endophytes among the tissues was evaluated using permutational multivariate analysis of variance (PERMANOVA).

Stacked bar plots generated in R (v3.3.1) were used to identify the most abundant bacterial communities at both the phylum and genus levels. Taxa with a relative abundance at the phylum level <1% were merged into other taxa, whereas taxa with a relative abundance at the genus level <3% were merged into other taxa.

Comparisons of taxonomic data at the phylum and genus levels among the five groups were performed using the Kruskal–Wallis test with post-hoc Tukey’s honest significant difference (HSD) test using the stats package in R (v3.3.1). Statistical significance was accepted as *p* < 0.05 (Segata et al., 2011).

Metagenomic functions were predicted using Phylogenetic Investigation of Communities by Reconstruction of Unobserved States 2 (PICRUSt2) based on representative OTU sequences. PICRUSt2 contains the following tools. HMMER was used to align OTU representative sequences with the reference sequences. EPA-NG and Gappa were used to place the representative OTU sequences into a reference tree. Castor was used to normalize the 16S gene copies. MinPath was used to predict the gene family profiles and locate gene pathways. The entire analysis process was performed according to PICRUSt2 protocols.

The online resource FUNGuild^2^ was used to parse the fungal community dataset from the rarefied OTU table into functional groups (or guilds). FUNGuild software annotates taxonomic data within the OTU table with corresponding data on its online database. The annotations include the guild, trophic mode, and growth morphology, and only confidence scores of “Probable” and “Highly Probable” were used (Douglas et al., 2020).

### 2.4 Determination of capsaicin in industrial chili peppers using high-performance liquid chromatography

#### 2.4.1 Capsaicin extraction from the sample

The fresh sample was cut with scissors, and 5 g of the fresh sample was crushed and mashed with a glass rod in a 50 mL centrifuge tube A. The sample was then dried at 50 °C until the moisture was reduced to less than 15%. Then, 12.5 mL of extraction solution [methanol: tetrahydrofuran = 1:1 (v:v)] was added, sealed with a plastic wrap, and a few small holes were made. The sample was subjected to sonication at 60 °C in a water bath for 30 min, and the clear liquid was transferred to another 50 mL centrifuge tube B. In centrifuge tube A, another 12.5 mL of extraction solution was added, sonicated at 60 °C in a water bath for 10 min, and the clear liquid was transferred to centrifuge tube B. The extraction was repeated. The centrifuge tube containing 37.5 mL of extract was placed in a 70 °C blast oven. The solution was concentrated to 5 mL, and then diluted to 15 mL using the extraction solution.

#### 2.4.2 Preparation of stock solution

The appropriate amount of capsaicin and dihydrocapsaicin was weighed and mixed with methanol to form a 1 mg/mL stock solution, which was sealed and stored in a refrigerator at 4 °C.

#### 2.4.3 Preparation for using the solution

The reserved solution was used to prepare 0, 20, 40, 60, 80, and 100 μg/mL control solutions, after sample injection to generate a standard curve.

#### 2.4.4 Instrumentation

High-performance liquid chromatography (HPLC) (Thermo Fisher VQ-CORE-QUAT-01) and UV detector (Mettler Toledo UV7).

#### 2.4.5 Chromatographic conditions

The chromatographic column included an Agilent SB-C18 (inner diameter 4.6 mm, length 150 mm, particle size 5 μm) in which the flow rate was 1.0 mL/min, the column temperature was 30 °C, the injection volume was 10 μL, the mobile phase was methanol:water = 65:35 (v:v), the collection time was 20 min, and the detection wavelength was 280 nm (Yang et al., 2024) (Table 1).

**Table 1:** Grouping and capsaicin content statistics.

Group	Sample number	Capsaicin content
td3	GYLJ_3	1.0186
	GYLJ_8	1.9358
	GYLJ_9	1.2135
	GYLJ_13	0.8505
	GYLJ_14	1.4262
td2	GYLJ_2	0.5194
	GYLJ_7	0.7365
	GYLJ_11	0.7051
	GYLJ_12	0.8087
	GYLJ_15	0.5649
td1	GYLJ_1	0.2673
	GYLJ_4	0.0772
	GYLJ_5	0.0979
	GYLJ_6	0.4726
	GYLJ_10	0.1712
sylj	SYLJ	0.0287

## 3 Results

### 3.1 Statistics of endophyte community sequencing results from different strains of industrial chili peppers

Tissue samples from different strains of industrial pepper plants were selected and sequenced for the endophytic bacterial 16S rRNA gene V5–V7 variable region and the endophytic fungal ITS rRNA gene. For the bacterial community, this sequencing generated 2,710,205 original sequences, with 2,677,023 valid sequences obtained after quality control, which were further clustered to obtain 10,936 ASVs based on a 97% similarity threshold, with an average sequencing coverage of 99.99%. In the fungal community, 3,669,711 original sequences were obtained, with 3,486,999 valid sequences obtained after quality control, and 2,593 ASVs were obtained after clustering, with an average sequencing coverage of 99.98%.

**Figure 1:**
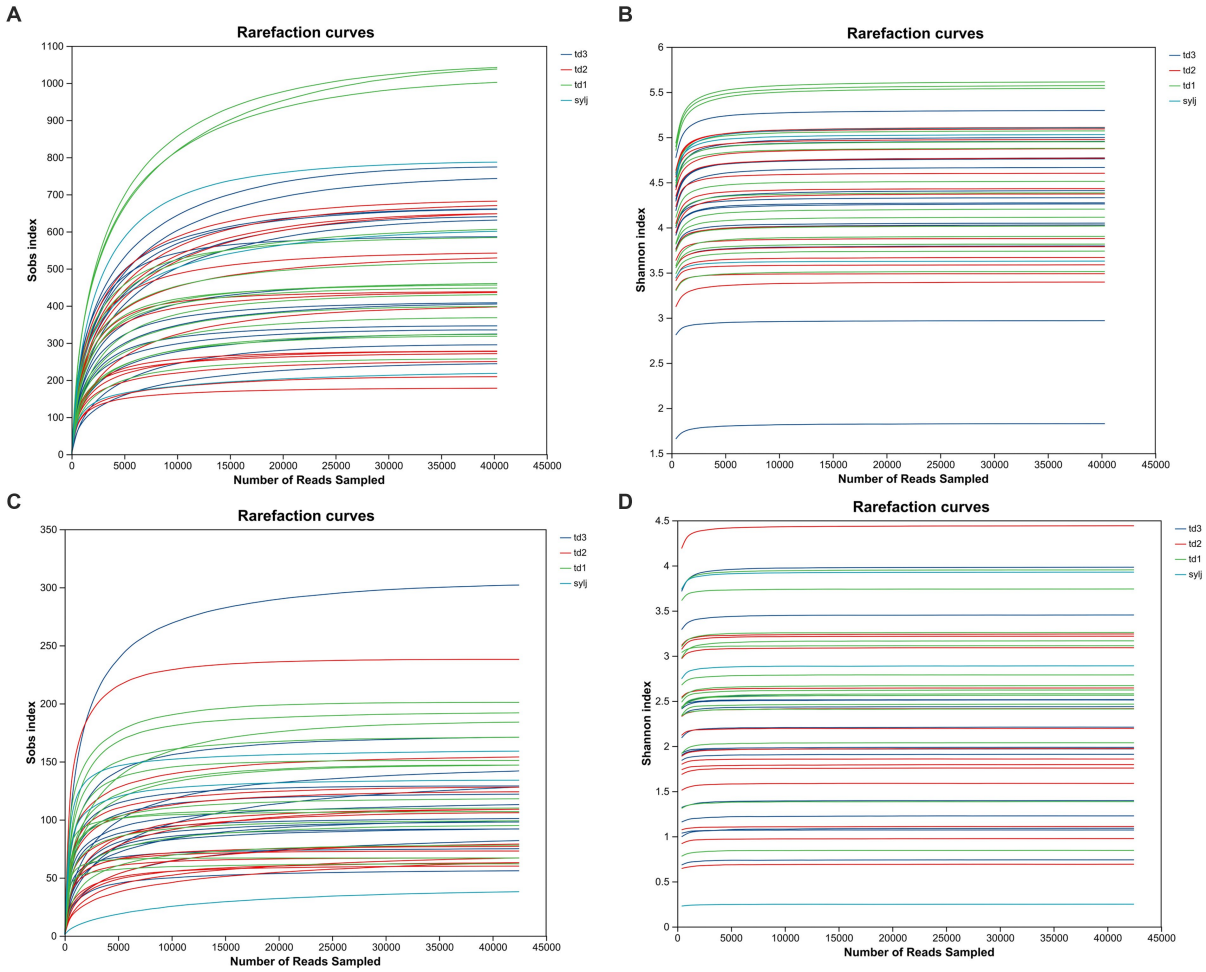
Alpha diversity index dilution plot of industrial chili pepper endophytes. **(A)** Dilution plot of the Sobs index in bacterial communities. **(B)** Dilution plot of the Shannon index in bacterial communities. **(C)** Dilution plot of Sobs index in fungal communities. **(D)** Dilution plot of the Shannon index in fungal communities.

For both bacterial and fungal communities, the Sobs community richness dilution curves and Shannon community diversity dilution curves for each sample tended to flatten, indicating that the amount of sequenced data was appropriate and sufficient to reflect most of the microbial diversity in the samples (Figure 1).

### 3.2 Diversity analysis of endophyte communities in different strains of industrial chili peppers

**Figure 2:**
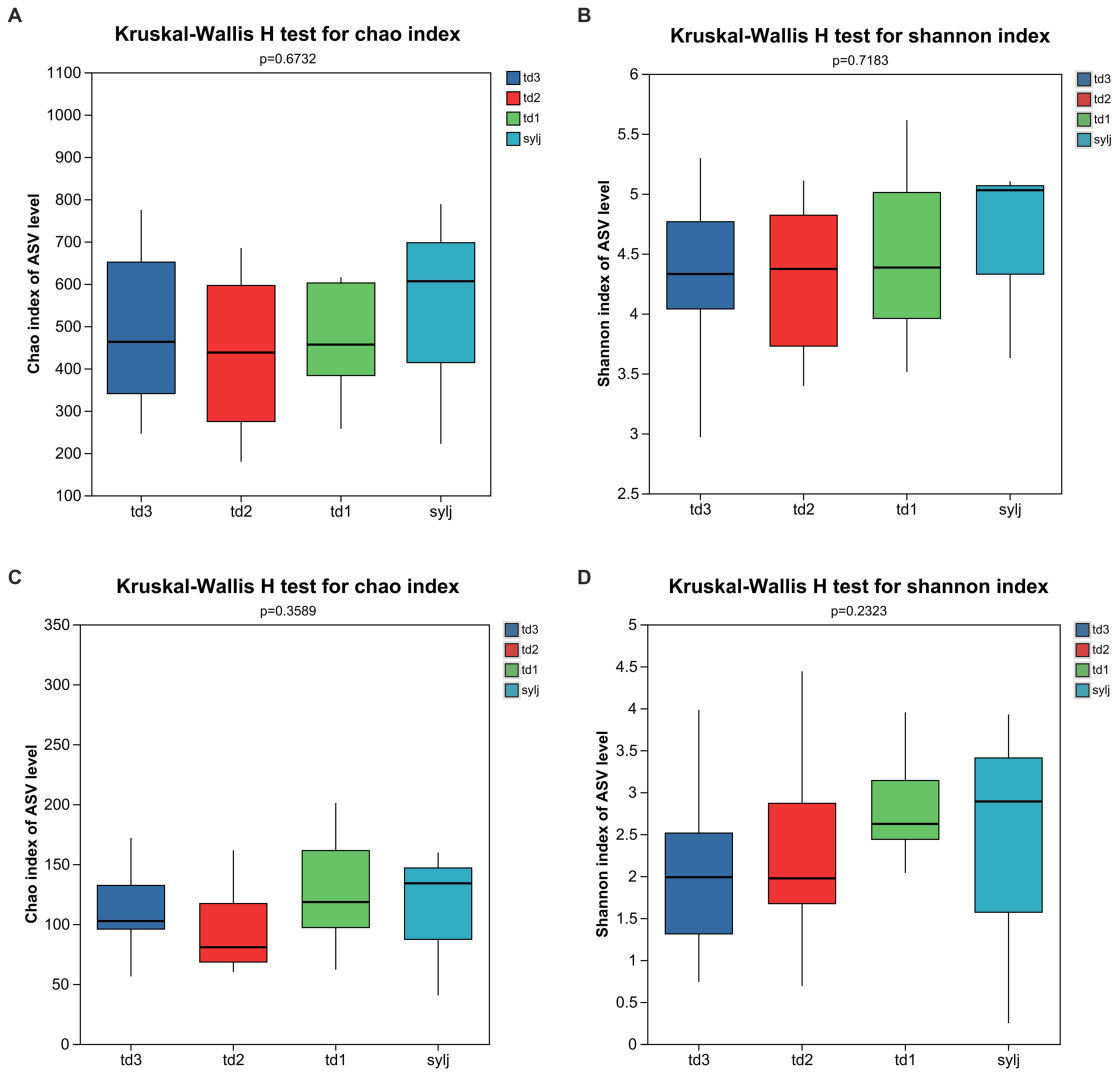
Comparison of endophyte alpha diversity indexes among different varieties of industrial chili peppers. **(A)** Test for differences in the bacterial community Chao index. **(B)** Test for differences in the bacterial community Shannon index. **(C)** Test for differences in the fungal community Chao index. **(D)** Test for differences in the fungal community Shannon index.

The Chao (*p* = 0.673) and Shannon indices (*p* = 0.718) of the bacterial community were not significantly different between groups, indicating no significant differences in endophytic bacterial abundance or diversity among the lines (Figures 2A,B). Tests of intergroup differences in the Chao (*p* = 0.358) and Shannon indices (*p* = 0.232) for the fungal community showed results similar to those for the bacterial community, with no significant differences in endophytic fungal abundance and diversity among the lines (Figures 2C,D).

**Figure 3:**
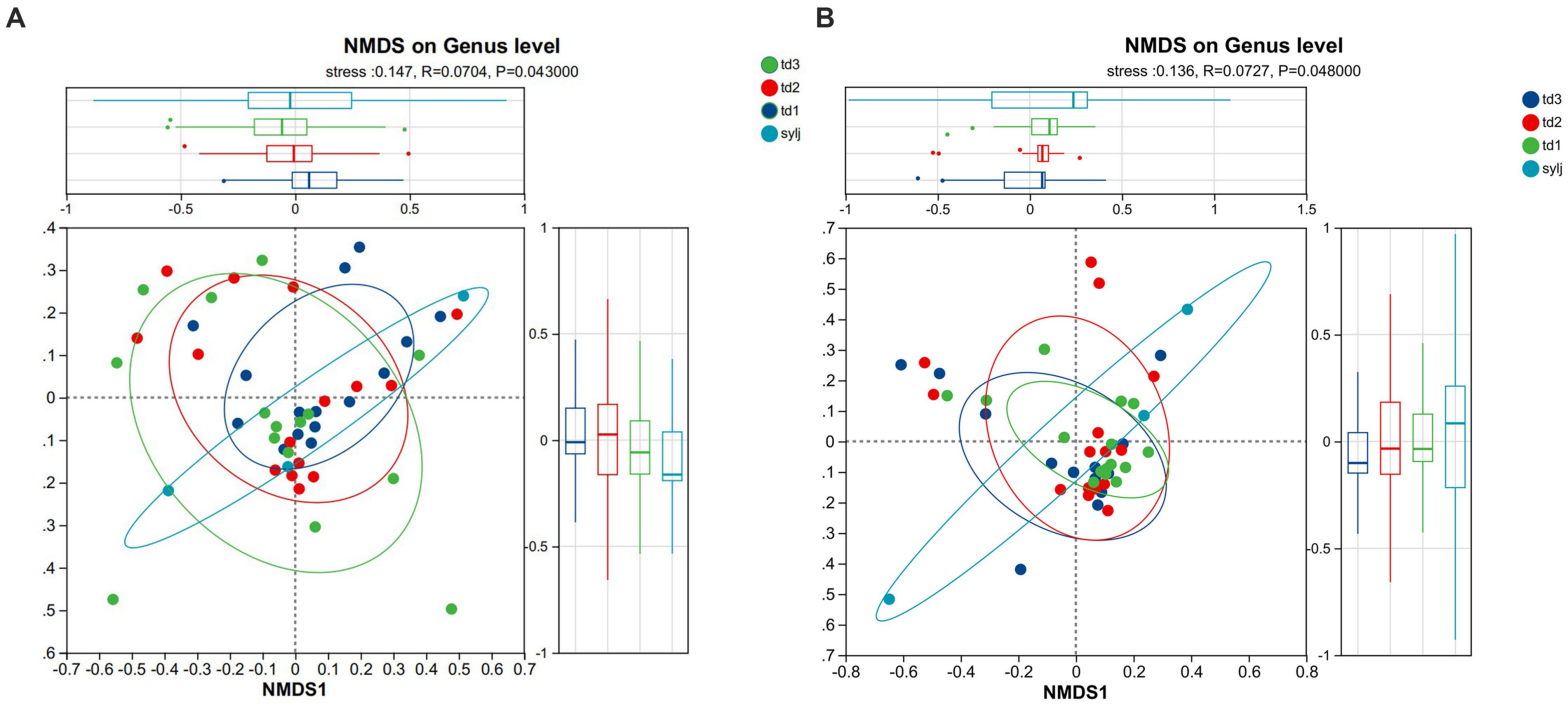
Non-metric multidimensional scaling (NMDS) analysis of endophytes among different varieties of industrial chili peppers. **(A)** NMDS analysis of the genus level in the bacterial community. **(B)** NMDS analysis of the genus level in the fungal community.

NMDS analysis of bacterial communities, based on the ANOSIM test for differences between groups (Figure 3A), showed that grouping had some significance in explaining the differences in bacterial community structure (stress = 0.147). It also indicated that differences between groups were greater than those within groups for different lines (*R* = 0.0704), and that bacterial community structure between groups was significantly different (*p* = 0.043). The results of the NMDS analysis of fungal communities (Figure 3B) showed that grouping had some significance in explaining the differences in colony structure (stress = 0.136), that the differences between-group were greater than the within-group differences in different strains (*R* = 0.0727), and that the structure of endophytic fungal communities in different groups was significantly different (*p* = 0.048).

### 3.3 Analysis of the endophytic bacterial flora composition in different strains of industrial chili peppers

**Figure 4:**
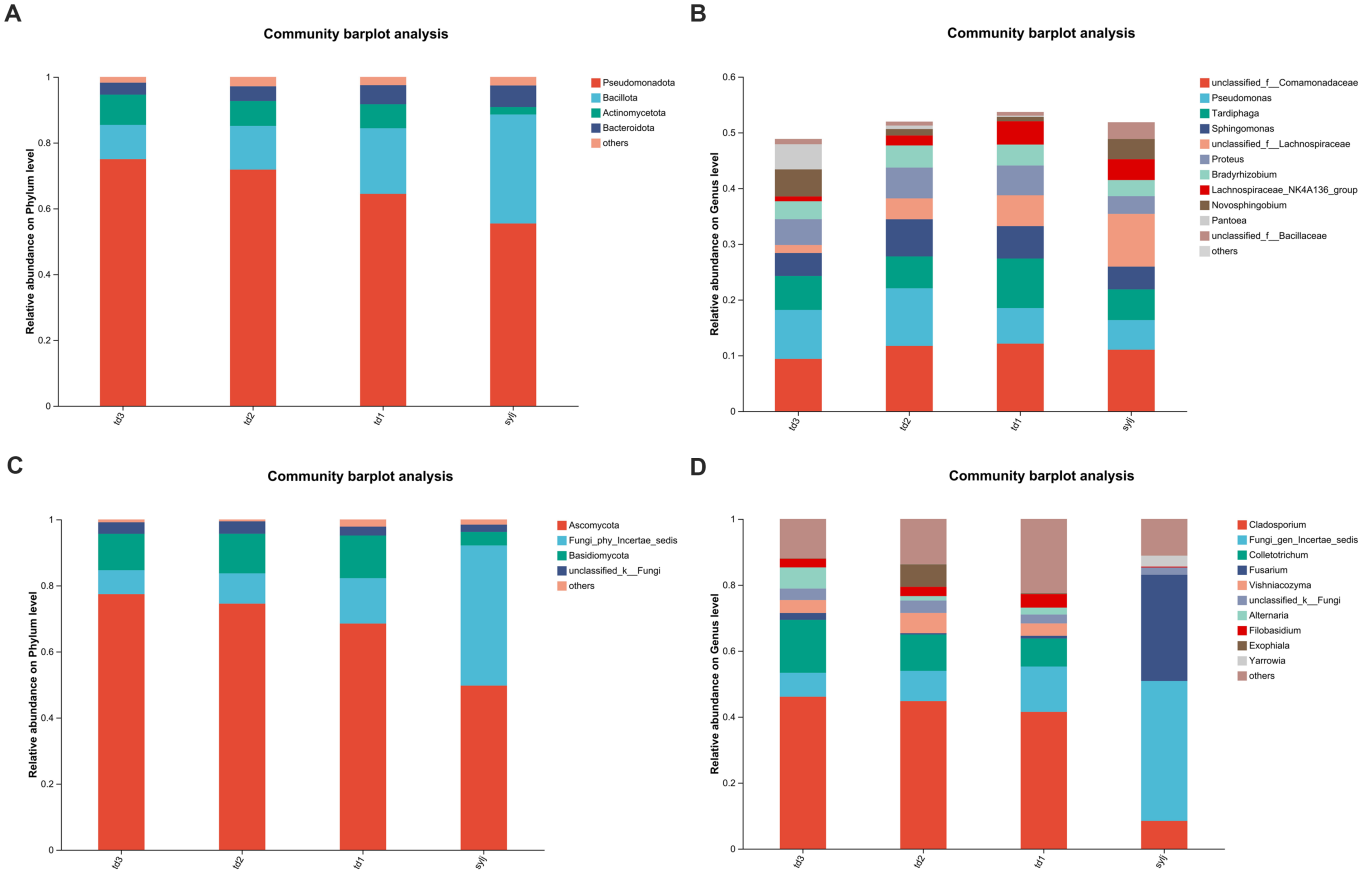
Analysis of endophyte community structure at the phylum and genus levels in different industrial chili pepper strains. **(A)** Community composition of endophytic bacteria at the phylum level. **(B)** Community composition of endophytic bacteria at the genus level. **(C)** Community composition of endophytic fungi at the phylum level. **(D)** Community composition of endophytic fungi at the genus level.

Overall, 10,936 endophytic bacterial amplicon sequence variants (ASVs) were compared among 43 phyla and 1,064 genera. After screening and combining phyla with less than 1% relative abundance share, at the phylum level (Figure 4A), *Pseudomonadota* was the predominant phylum in all four groups: td1, td2, td3, and sylj (64.45, 71.83, 75, and 55.45%, respectively). This was followed by Bacillota, which was noteworthy in that the percentage of this phylum in sylj (33.12%) was much larger than that in the other three groups. There was little difference in the percentage of this phylum in td1, td2, and td3 (19.95, 13.26, and 10.41%, respectively). The third most dominant phylum in all three groups of industrial chili peppers was Actinomycetota (7.28, 7.57, 9.19%), followed by Bacteroidota (5.79, 4.46, and 3.64%). In the Sylj subgroup, Bacteroidota (6.55%) was more abundant than Actinomycetota (2.26%). At the genus level (Figure 4B), td1 samples from unclassified__f__Comamonadaceae (12.12%), *Tardiphaga* (8.88%), *Pseudomonas* (6.39%), *Sphingomonas* (5.82%), unclassified__f__ Lachnospiraceae (5.53%), *Proteus* (5.35%). Eight other bacterial genera showed a relative abundance percentage greater than 3%. In td2 samples, seven bacterial genera, including unclassified_f__Comamonadaceae (11.71%), *Pseudomonas* (10.34%), *Sphingomonas* (6.67%), *Tardiphaga* (5.71%), and *Proteus* (5.53%), had a relative abundance greater than 3%. In td3 samples, unclassified_f__Comamonadaceae (9.38%), *Pseudomonas* (8.79%), *Tardiphaga* (6.09%), *Novosphingobium* (4.85%), *Proteus* (4.63%), *Pantoea* (4.51%), and eight bacterial genera had relative abundances greater than 3%. In the sylj group, classified __f__Comamonadaceae (11.03%), unclassified__f__Lachnospiraceae (9.46%), *Tardiphaga* (5.51%), *Pseudomonas* (5.31%), *Sphingomonas* (4.09%), and nine other bacterial genera showed relative abundances greater than 3%.

A total of 2,593 endophytic fungal ASVs were compared with 17 phyla and 652 genera. After screening and merging the phyla with less than 1% share at the phylum level (Figure 4C), *Ascomycota* was found to be predominant in all four subgroups of td1, td2, td3, and sylj (68.49, 74.52, 77.39, and 49.71%, respectively). The relative abundance of Basidiomycota (11.98 and 11.02%) was greater than that of ngi_phy_Incertae_sedis (9.17 and 7.25%) in td2 and td3, respectively. In the td1 and sylj subgroups, the opposite was true, with ngi_phy_Incertae_sedis (13.75 and 42.43%, respectively) showing a greater relative abundance than that of Basidiomycota (12.90 and 4.09%, respectively). The phylum with the fourth-highest relative abundance, unclassified_k_fungi, was exceptionally uniform in all four subgroups (td1, 2.79%; td2, 3.76%; td3, 3.48%; sylj, 2.20%). Notably, Mortierellomycota (1.66%) showed a relative abundance greater than 1% only in td1, and Olpidiomycota had a relative abundance greater than 1% only in the sylj group. At the genus level (Figure 4D), td1, td2, and td3 had similar endophytic community compositions, whereas sylj differed significantly from these three subgroups. Specifically, five genera, *Cladosporium* (41.51%), Fungi_gen_Incertae_sedis (13.75%), *Colletotrichum* (8.50%), and *Filobasidium* (4.10%) had relative abundances greater than 3% in td1. In td2, *Cladosporium* (44.77%), *Colletotrichum* (11.02%), Fungi_gen_Incertae_sedis (9.17%), *Exophiala* (6.84%), and *Vishniacozyma* (6.13%) had relative abundances greater than 3%. In td3, six genera of bacteria, *Cladosporium* (46.10%), *Colletotrichum* (16.05%), Fungi_gen_Incertae_sedis (7.25%), and *Alternaria* (6.41%) had relative abundances greater than 3%. In the sylj subgroup, Fungi_gen_ Incertae_sedis (42.43%), *Fusarium* (32.19%), *Cladosporium* (8.45%), and *Yarrowia* (3.32%), accounted for more than 3% of the relative abundance.

### 3.4 Analysis of differences in the endophyte communities of different strains of industrial chili peppers

**Figure 5:**
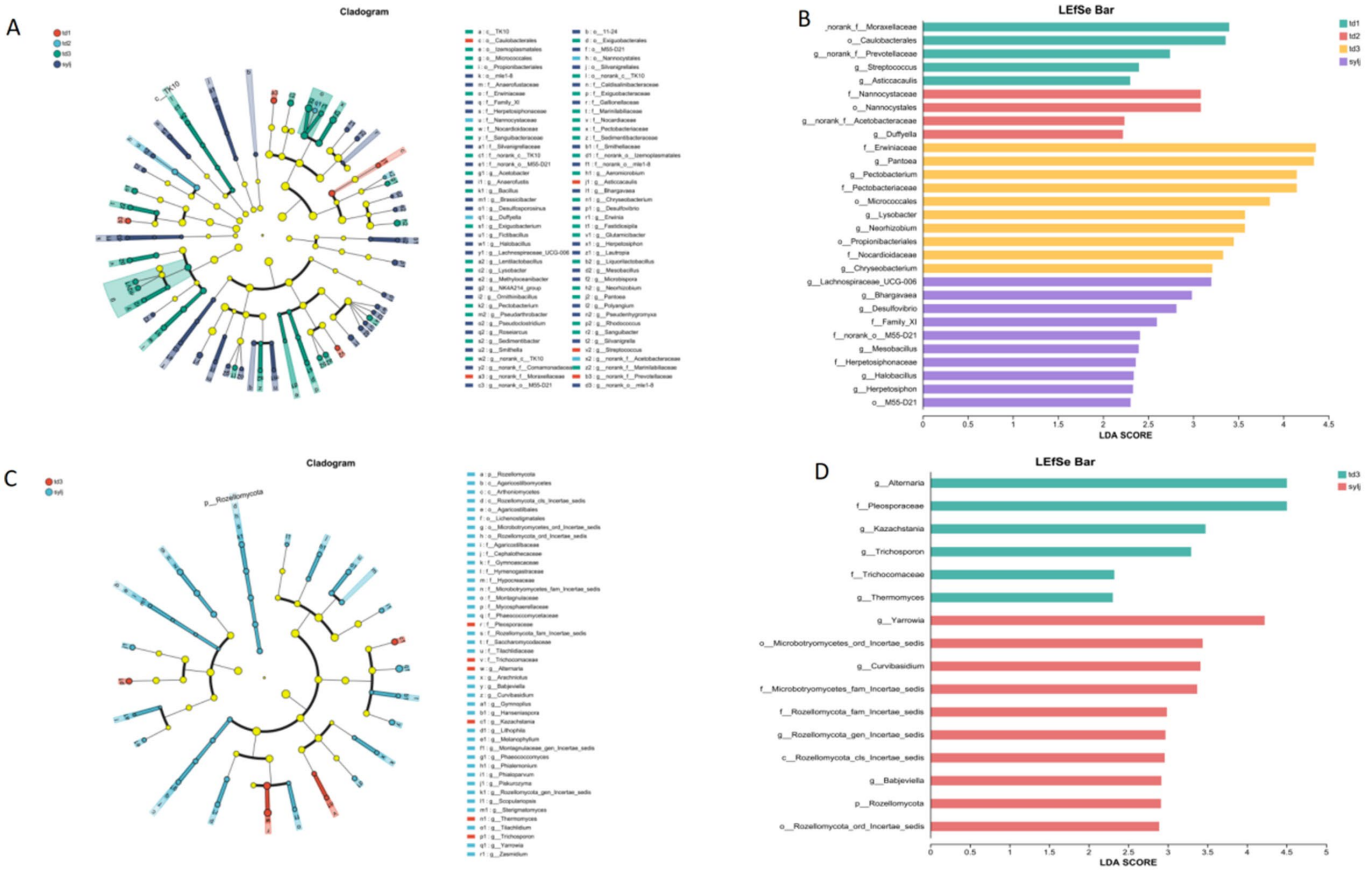
Analysis of endophyte community differences in different industrial chili peppers strains. **(A)** Linear discriminant analysis Effect Size (LEfSe) for multilevel species differences in bacterial communities. **(B)** Linear discriminant analysis (LDA) of bacterial communities. **(C)** LEfSe for multilevel species differences in fungal communities. **(D)** LDA of fungal communities.

**Figure 6:**
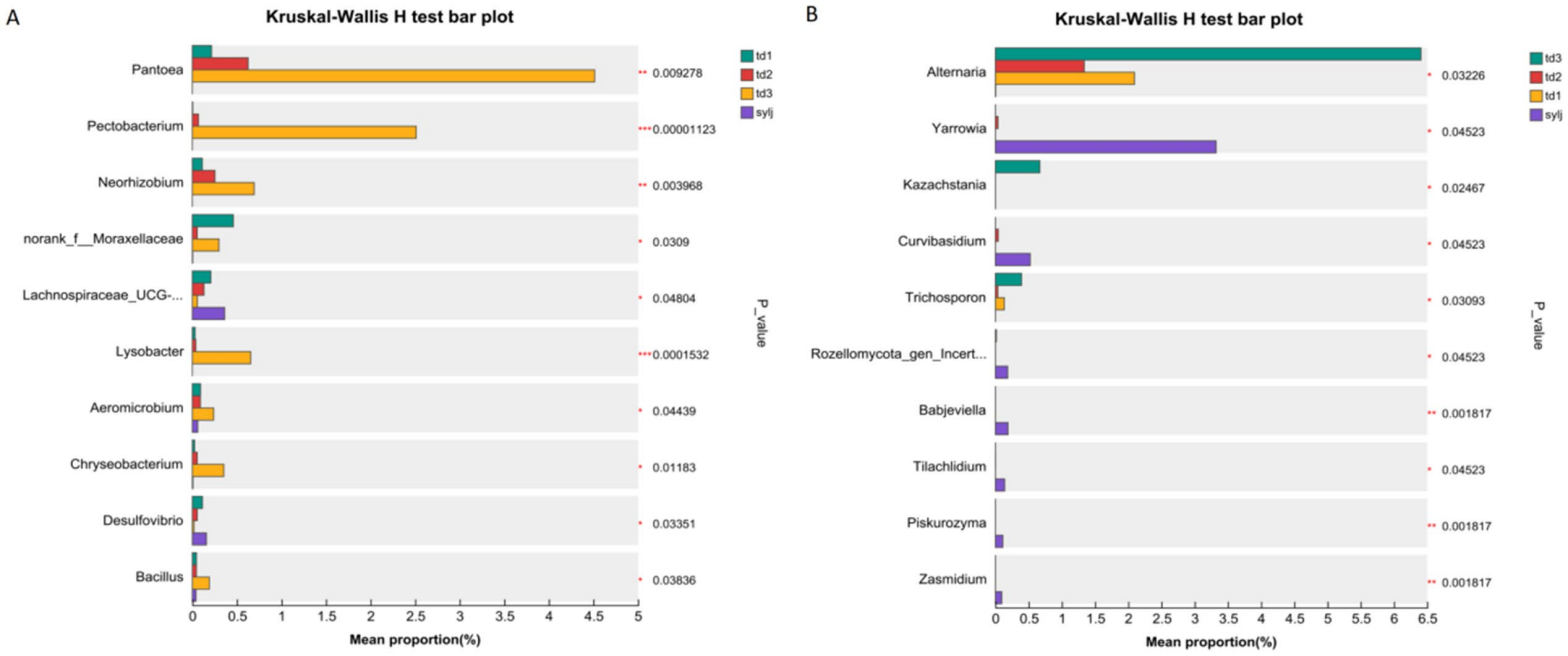
Tests for intergroup differences in different strains of industrial chili pepper endophytes. **(A)** Tests for differences in bacterial communities. **(B)** Tests for differences in fungal communities.

The LEfSe analysis for multilevel species differences showed that at the bacterial community phylum level (Figure 5A), no phyla with significant differences were observed in the samples. At the genus level, five bacterial genera, including *Asticcacaulis* and *Caulobacterales*, were significantly enriched in the td1 samples. Four genera, including *Nannocystaceae* and *Duffyella*, were enriched in the td2 samples. The td3 and syjl subgroups contained more genera that were significantly enriched, and 20 genera, including *Micrococcales* and *Acetobacter*, were significantly enriched in td3. The sylj sample was significantly enriched in 24 genera, including *Anaerofustis* and *Brassicibacter*. Linear discriminant analysis (LDA) showed that the endophytic bacterial genera (Figure 5B) were significantly enriched in the td1 and td2 samples in the LEfSe analysis, which had a significant effect on the differences between strains (LDA > 2). Of the 20 genera significantly enriched in td3 samples, 10 had a considerable impact on the differences among strains. Further, among 24 genera significantly enriched in the sylj sample, 10 significantly affected the differences between strains. The results of the tests for intergroup differences in bacterial communities (Figure 6A) showed that 10 genera, led by *Pantoea*, *Pectobacterium*, and *Neorhizobium*, significantly affected the colony structure of the td1, td2, td3, and sylj samples (*p* < 0.05).

The results of LEfSe multilevel species hierarchical analysis of the fungal community (Figure 5C) showed significant differences in endophytic fungal enrichment between the td3 and sylj samples. At the phylum level, Rozellomycota was significantly enriched in the sylj samples. At the genus level, *Alternaria*, *Kazachstania*, *Thermomyces*, and *Trichospoporon* were enriched in td3 samples. Eighteen genera of fungi, including *Arachniotus*, *Babjeviella*, and *Curvibasidium*, were significantly enriched in sylj samples. LDA showed that all endophytic fungi (Figure 5D) significantly enriched in the td1 sample, as indicated by the LEfSe analysis, significantly affected the differences between the lines (LDA >2). Further, 10 of the 18 genera significantly enriched in the sylj sample significantly affected the differences between lines. The tests for intergroup differences in fungal communities (Figure 6B) showed that 10 genera led by *Alternaria*, *Yarrowia*, and *Kazachstania* had a significant effect on the colony structure of td1, td2, td3, and sylj samples (*p* < 0.05) (see Figure 6).

### 3.5 Analysis of the functional composition of endophytic bacterial flora among different lines of industrial chili peppers

**Figure 7:**
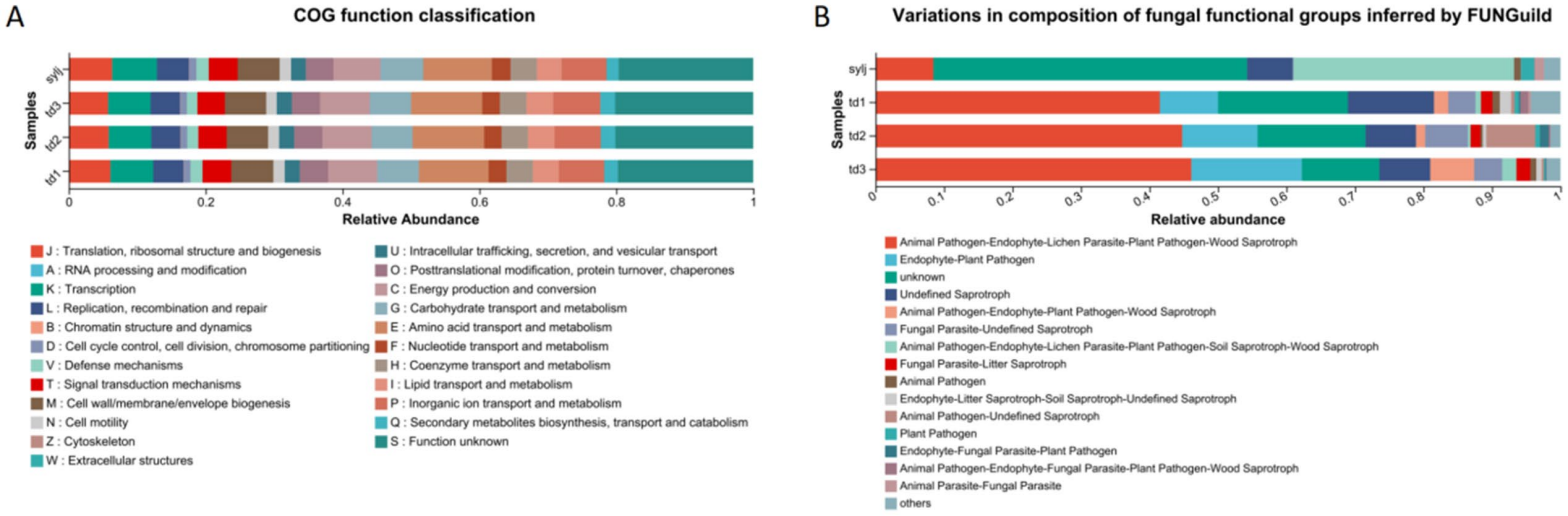
Prediction analysis of endophyte function in industrial chili peppers. **(A)** Bacterial Clusters of Orthologous Genes (COG) function prediction. **(B)** Fungal FUNGuild function prediction.

Twenty-four COG functions were obtained by comparing the bacterial EggNOG database. The analysis of COG functional composition among the lines showed that the top 10 COG functions in the td1 samples were, in order: function unknown (S, 19.77%), amino acid transport and metabolism (E, 10.18%), energy production and conversion (C, 7.18%), inorganic ion transport and metabolism (P, 6.62%), transcription (K, 6.24%), cell wall/membrane/envelope biogenesis (M, 6.14%), carbohydrate transport and metabolism (G, 6.09%), translation, ribosomal structure, and biogenesis (J, 6.07%), replication, recombination, and repair (L, 4.42%), and signal transduction mechanisms (T, 4.21%). The top 10 COG functions in td2 were S (20.14%), E (10.45%), C (7.21%), P (6.74%), K (6.21%), M (6.06%), G (5.97%), J (5.82%), L (4.26%), and T (4.13%). Those in td3 were S (20.16%), E (10.39%), C (7.27%), P (6.88%), K (6.18%), G (6.032%), M (6.031%), J (5.74%), L (4.31%), and O (post-translational modification, protein turnover, chaperones, 4.12%). The top 10 COG functions in the sylj sample were S (19.65%), E (10.02%), C (6.86%), P (6.55%), K (6.53%), J (6.33%), G (6.26%), M (6.12%), L (4.68%), and T (4.24%) (Figure 7).

The FUNGuild analysis of endophytic fungi showed that the fungal trophic pattern Animal undefined Pathogen-Endophyte-Lichen Parasite-Plant Pathogen-Wood Saprotroph (42.74, 45.86, and 47.16%, respectively) had the highest percentage in td1, td2, and td3 samples. Further ranking of the trophic mode share in td1 samples included unknown (16.76%), Undefined Saprotroph (13.47%), and Endophyte-Plant Pathogen (7.48%). In td2, the subsequent fungal trophic patterns were unknown (18.57%), Endophyte-Plant Pathogen (9.67%), and Undefined Saprotroph (7.17%). The remaining trophic patterns in td3 were Endophyte-Plant Pathogen (19.13%), unknown (10.31%), and Undefined Saprotroph (7.48%). The trophic pattern composition of the sylj sample differed significantly from that of the other three samples, with unknown (48.57%) showing the largest proportion, followed by Animal Pathogen-Endophyte-Lichen Parasite-Plant Pathogen-Soil Saprotroph-Wood Saprotroph (43.51%), which was the largest among the other three samples. Animal Pathogen-Endophyte-Lichen Parasite-Plant Pathogen-Wood Saprotroph accounted for only 8.57% in the sylj samples.

## 4 Discussion

Regarding the diversity and community composition of endophytes in industrial chili peppers, although the diversity of endophyte communities was consistent among lines, NMDS analysis based on the ANOSIM test of variance indicated a specific endophyte community structure among lines. A significant difference was observed in community composition between the high capsaicin concentration industrial chili subgroup (td3) and the low capsaicin concentration edible chili subgroup (sylj), whereas the community composition of td3 differed compared to that of the medium concentration industrial chili subgroup (td2) but not as significantly as the difference between td3 and sylj. Since all experimental subjects were chili peppers with the same management practices and growing habitats, this result may be attributed to the differences in capsaicin concentrations among the strains. In other words, the different capsaicin concentrations may have altered the internal environment of chili peppers, leading to the creation of a relatively specific and stable colony structure among different chili peppers (Zhang et al., 2024). Previous findings in other plants have also shown that during the long-term co-evolutionary process between host plants and endophytes, plant endophytes have adapted to specific host internal environments, and their species, distribution, and colonization abilities vary depending on the internal habitats of the host plants (Wen et al., 2004). This has led to the development of host-specific characteristics of endophytes in the same plant species. For example, the diversity of endophytes in *Astragalus* (Wen et al., 2004), *Codonopsis* (Shi et al., 2006), Forsythia (Yun et al., 2010), *Seabuckthorn* (Zhang et al., 2018), and Soybean (Qu et al., 2023) differs significantly among themselves. Furthermore, endophyte communities in the same species of plants differ significantly because of differences in the plant growth habitats and genetic characteristics (Strobel, 2003). The reason is that the habitat changes the internal environment of the host and thus shapes the endophyte communities. This specificity in endophyte community composition results in host plants showing faster growth rates, greater resistance to adversity and disease, and greater resistance to animal predation (Nguyen et al., 2016).

In analyzing the microbial community structure within industrial peppers, we found similarities with other plants studied for endophytic microbes. The dominant phylum of endophytic microorganisms in industrial peppers was generally consistent in the species of dominant phyla. However, differences were observed in the relative abundance among different strains of industrial peppers. The dominant bacterial phylum was *Pseudomonadota*, whereas the dominant fungal phylum was *Ascomycota*, a general commonality with the endophytic bacteria from other plants at the phylum level. In terms of species composition at the phylum level, the three subgroups of industrial chili peppers (td1, td2, and td3) were essentially identical, whereas the phylum-level community composition of the edible chili peppers subgroup (sylj) differed greatly from that of the remaining three subgroups, most likely because of the differences in capsaicinoid concentrations, as all four subgroups were planted and managed consistently. Notably, many microorganisms belonging to *Pseudomonadota* produce indole acetic acid, gibberellins, and different types of antibiotics, which are essential for promoting plant growth, suppressing pathogens, and boosting plant disease resistance. This may be beneficial for the adaptation of industrial chili peppers to hostile high-altitude environments (Picard et al., 2000; Ramesh et al., 2009). At a more specific level of bacterial genera, the proportion of shared microbial genera spanning different lineages in all subgroups exceeded 90%, emphasizing the importance of these shared microbial genera in constituting the basic microbial community. However, fungal genera were more responsive to capsaicin concentrations than bacteria, with their dominant genus*, Cladosporium*, showing a tendency to decrease with increasing capsaicin concentrations in all three subgroups of industrial chili peppers. The fungal community composition in the edible pepper subgroup was approximately the same as that in the other three subgroups; however, the relative abundance percentages were significantly different. Furthermore, many unidentified species were found in the endophytic fungal community composition, indicating the presence of a large endophytic fungal resource in industrial chili peppers that could be further developed.

Community variance analysis revealed significant differences in the bacterial communities of the four different pepper lines, which showed significantly different enrichments of genera. In contrast, fungi were differentially enriched only in the td1 line with a high capsaicin concentration and in the sylj line with a low capsaicin concentration. The presence of specific microbial genera in particular lines may reflect the specialized roles of these microbial genera in specific functions or physiological processes in different lines. For example, *Bacillus*, *Exiguobacterium*, *Chryseobacterium*, *Tilachlidium*, and *Zasmidium*, which are predominantly enriched in the high-capsaicin-concentration industrial chili subgroup (td3), have all been reported to perform important functions in various physiological and biochemical activities in host plants. The roles of *Bacillus* and *Chryseobacterium* include, but are not limited to, producing pro-biotic hormones, promoting plant nutrient uptake, enhancing secondary metabolite production, increasing plant photosynthesis, secreting antimicrobial substances, and inducing plant disease resistance. *Bacillus* itself has a broad-spectrum inhibitory property, which is indispensable to the plant in a variety of adverse stresses (Ordentlich, 1988; Hameeda et al., 2008). *Exiguobacterium* produces antioxidant enzymes and low-temperature adaptive proteins to promote the growth of cold plants and enhance frost resistance (Kasana and Pandey, 2018). *Tilachlidium* and *Zasmidium* produce indole alkaloids, promote the production of secondary metabolites, inhibit the growth of phytopathogenic bacteria, and enhance host resistance to fungal diseases (Mains, 1951; Aviles-Noriega et al., 2024). Although these strains have been shown to exert important promotional effects on physiological and biochemical processes in the host plant, their specific functions in industrial chili peppers remain unexplored. Thus, future studies need to focus on whether these strains promote the production of secondary metabolites such as capsaicin.

Plant endophytes have a relatively stable internal environment because the plant protects them, and endophytic bacteria are involved in various plant life activities through lateral gene transfer with the host plant (Zhi-Lin et al., 2008; Xuguang et al., 2012). Endophytic fungi, during their long-term evolution within the host, are equipped with survival strategies that switch between symbiosis, parasitism, and saprophytism to cope with different physiological states of the host plant and play important ecological roles in this phenotypic plasticity (Picard et al., 2000; Xu et al., 2021). In the present study, the endophytic bacteria of industrial chili peppers showed a rich array of metabolic functions, with genes related to unknown functions accounting for the largest proportion of all four subgroups. Furthermore, amino acid transport and metabolism (E), energy production and conversion (C), and inorganic ion transport and metabolism (P) were heavily enriched in all four samples. Enrichment of these genes provides a basic guarantee for the growth and development of industrial chili peppers, while cell wall/membrane/envelope biogenesis (M), translation, ribosomal structure and biogenesis (J), and replication, recombination and repair (L) are all highly enriched in the four samples, possibly because of the high altitude and strong radiation ecological environment of the Tibetan Plateau. This is prone to damage plant tissues, in turn prompting significant enrichment of endophytic bacterial gene resources involved in cellular processes related to the plant tissues of industrial peppers to help plants withstand the damage and stress of extreme environments (Ramesh et al., 2009). Furthermore, a certain percentage of genes related to translation, ribosomal structure, and biogenesis are also found in industrial peppers, suggesting that endophytic bacteria may participate in host plant metabolism. Regarding endophytic fungi, the largest proportion of unknown trophic types in the trophic patterns of different strains may be attributed to the incomplete analysis of the FUNGuild database (Igarashi et al., 2006). Simultaneously, it also reflects a large number of unknown endophytic fungal resources to be explored in extremely spicy industrial chili peppers. Based on the insurance effect theory of biodiversity, the more diverse the functional trophic types of endophytic fungi, the greater is the possibility that endophytic fungi will undertake more ecological functions for the host plant under new environmental conditions (Hameeda et al., 2008). Different functional trophic types in industrial chili peppers help the host adapt to the external environment, and differential mycorrhizal functions among strains enable endophytic fungi to perform specific functions in different strains of the host plant. Overall, by minimizing external interference in the experiments, it is highly likely that the differences in the function of some endophytic bacteria or the trophic phenotype of endophytic fungi are influenced by the variations in capsaicinoid concentrations among different strains of industrial chili peppers.

## 5 Conclusion

In this study, we analyzed the community composition and diversity of endophytes in extremely hot industrial chili peppers cultivated in Tibet using high-throughput sequencing technology to provide a reference for the in-depth exploration of endophyte resources and efficient cultivation of industrial chili peppers on the Tibetan Plateau. No significant differences in endophyte diversity were observed among the lines; however, due to variation in capsaicinoid concentrations, significant differences in the community composition were observed between the high capsaicinoid concentration industrial chili group (td3) and the low capsaicinoid concentration edible chili group (sylj). Although differences were found in the community composition of td3 compared to the higher concentration industrial chili group (td2), these differences were not as significant as those between td3 and sylj. The NMDS analysis, based on the ANOSIM test of variance, showed significant differences in bacterial and fungal community composition among the lines. The results of community composition analysis indicated that the dominant phylum of endophytic microorganisms in industrial chili peppers was generally consistent with the species of the dominant phyla. Although differences were seen in the relative abundance percentage, the dominant bacterial and fungal phyla were *Pseudomonadota* and *Ascomycota*, respectively. At the bacterial phylum level, the abundance percentage of different strains within shared microbial phyla across all subgroups was more than 90%. Fungi were more responsive to capsaicin concentration compared to bacteria, leading to variation in community composition among the strains. The results of community variance analysis showed that the bacterial communities of the four different pepper lines showed significantly differences in the enrichment of genera, whereas the fungi showed differential enrichment only in the td1 line with high capsaicin concentration and the sylj line with low capsaicin concentration. The functional abundance statistics of endophytic bacterial COG revealed that genes related to unknown functions made up the largest proportion in all four subgroups. Additionally, the functional prediction results of fungi using FUNGuild indicated the largest proportion of unknown nutrients in the nutritional pattern. Both bacterial and fungal functional predictions revealed genes and functions that contributed to plant growth and development. Moreover, many unidentified strains and unknown functions were found in this study, implying a large number of endophytic bacterial resources for further development and utilization in extremely hot industrial chili peppers. Overall, the difference in capsaicin concentration among the industrial chili strains affected the community structure and composition of endophytes. These findings provide a foundation for the isolation and identification of beneficial microorganisms from industrial chili peppers in the future. They also offer theoretical support for the efficient cultivation and popularization of extremely hot industrial chili peppers in Tibet.

## Data Availability

The datasets presented in this study can be found in online repositories. The names of the repository/repositories and accession number(s) can be found at: https://www.ncbi.nlm.nih.gov/, the accession number PRJNA1259232.

## Footnotes

^1^https://www.i-sanger.com/

^2^http://www.funguild.org/
