# Supplementary figures and images for: Analysis of the community composition and diversity of endophytes in extremely spicy industrial chili peppers from Tibet using high-throughput sequencing
Source: Front Microbiol. 2025 Sep 1;16:1630090. doi: 10.3389/fmicb.2025.1630090 (PMC12434037; doi:10.3389/fmicb.2025.1630090)

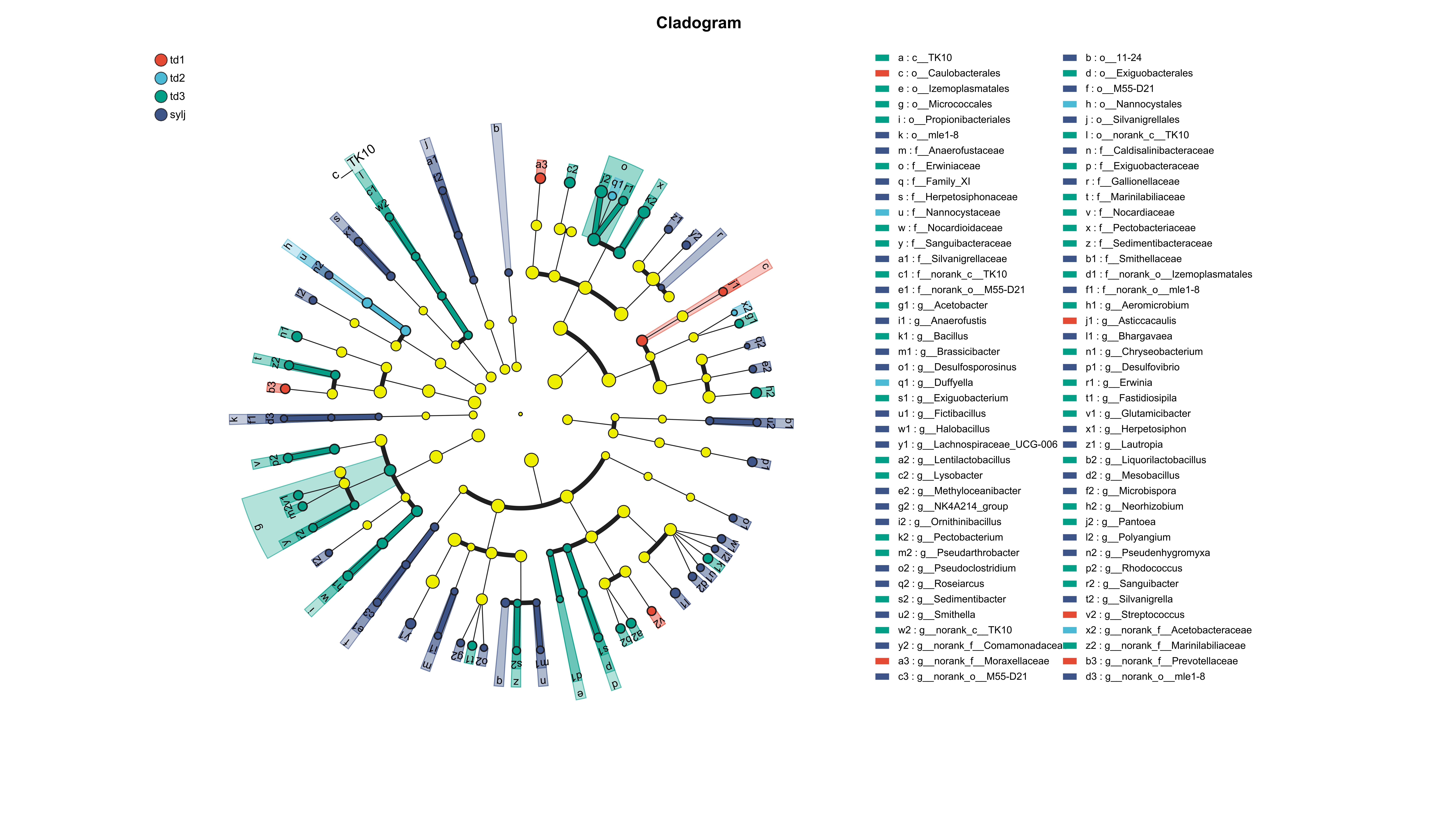

Supplement: Supplementary file 1 [file Image_1.JPEG]

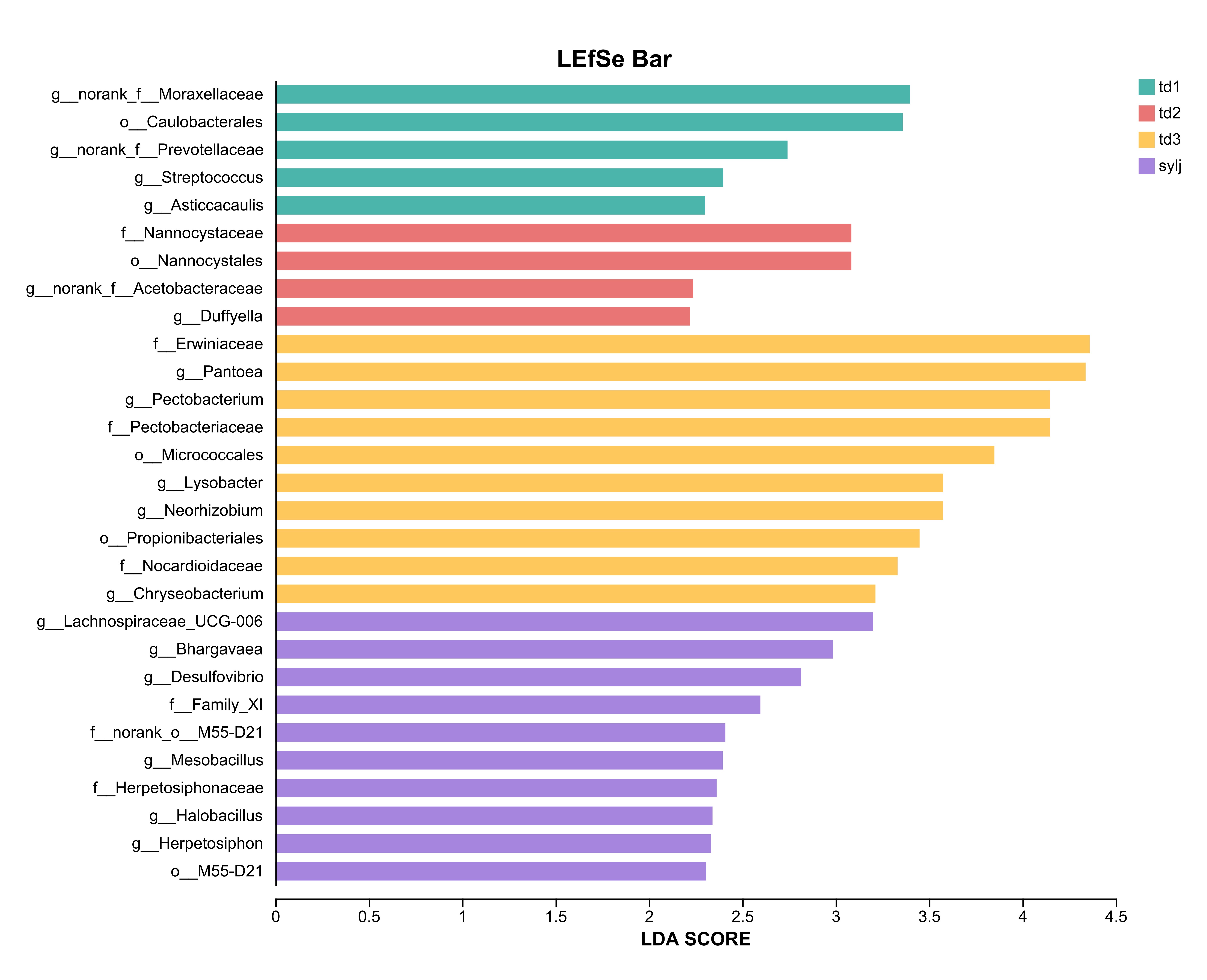

Supplement: Supplementary file 2 [file Image_2.JPEG]

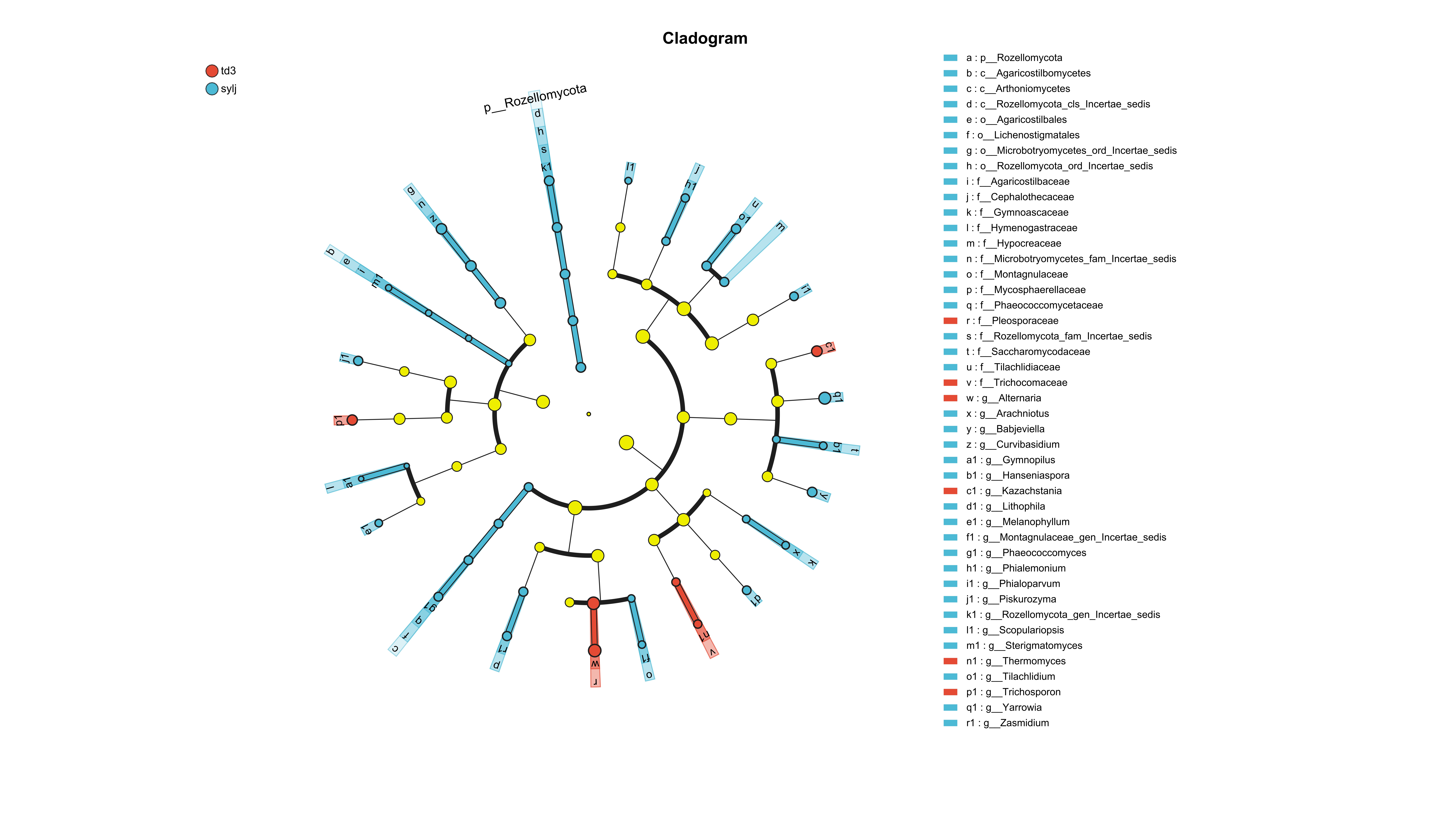

Supplement: Supplementary file 3 [file Image_3.JPEG]

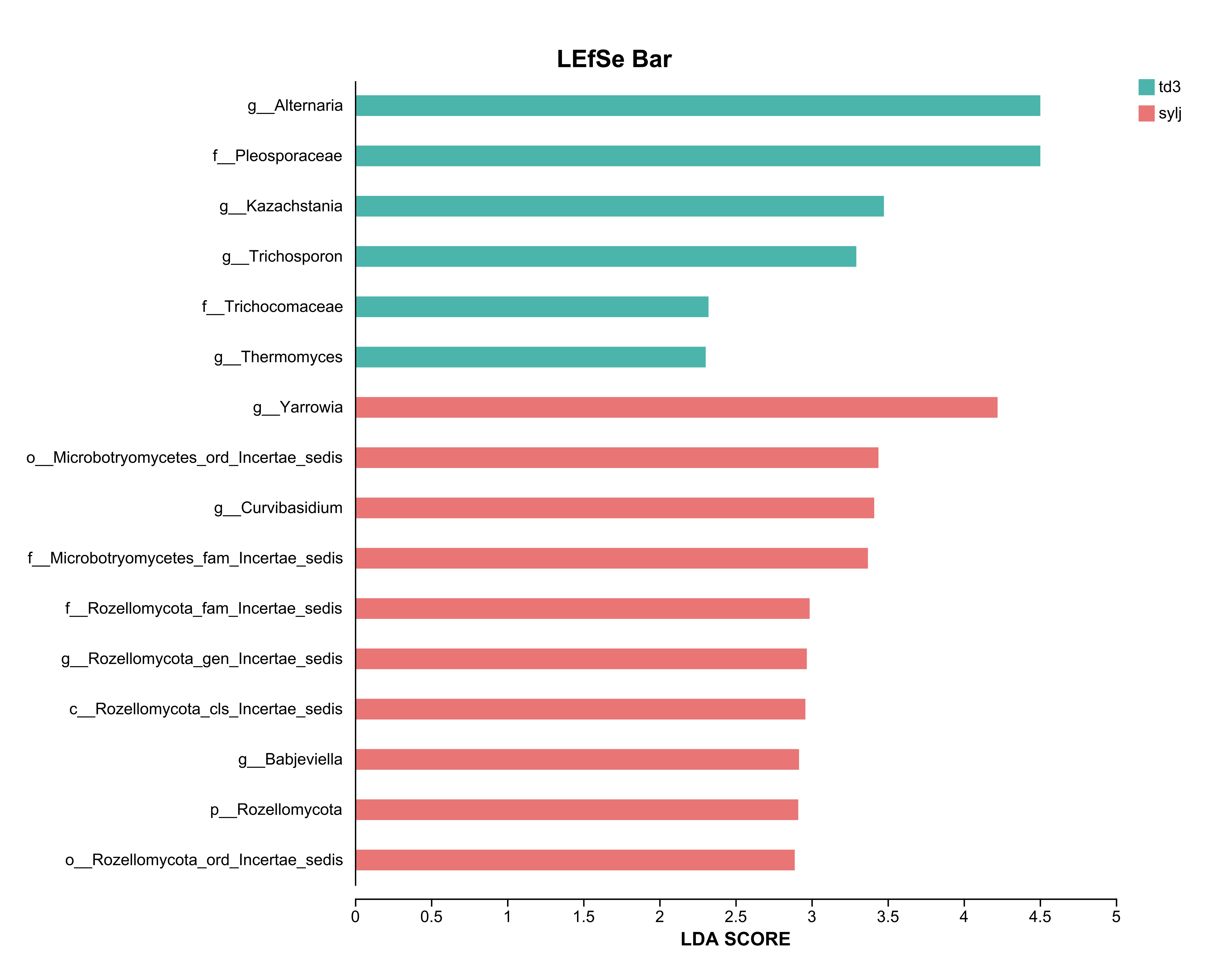

Supplement: Supplementary file 4 [file Image_4.JPEG]

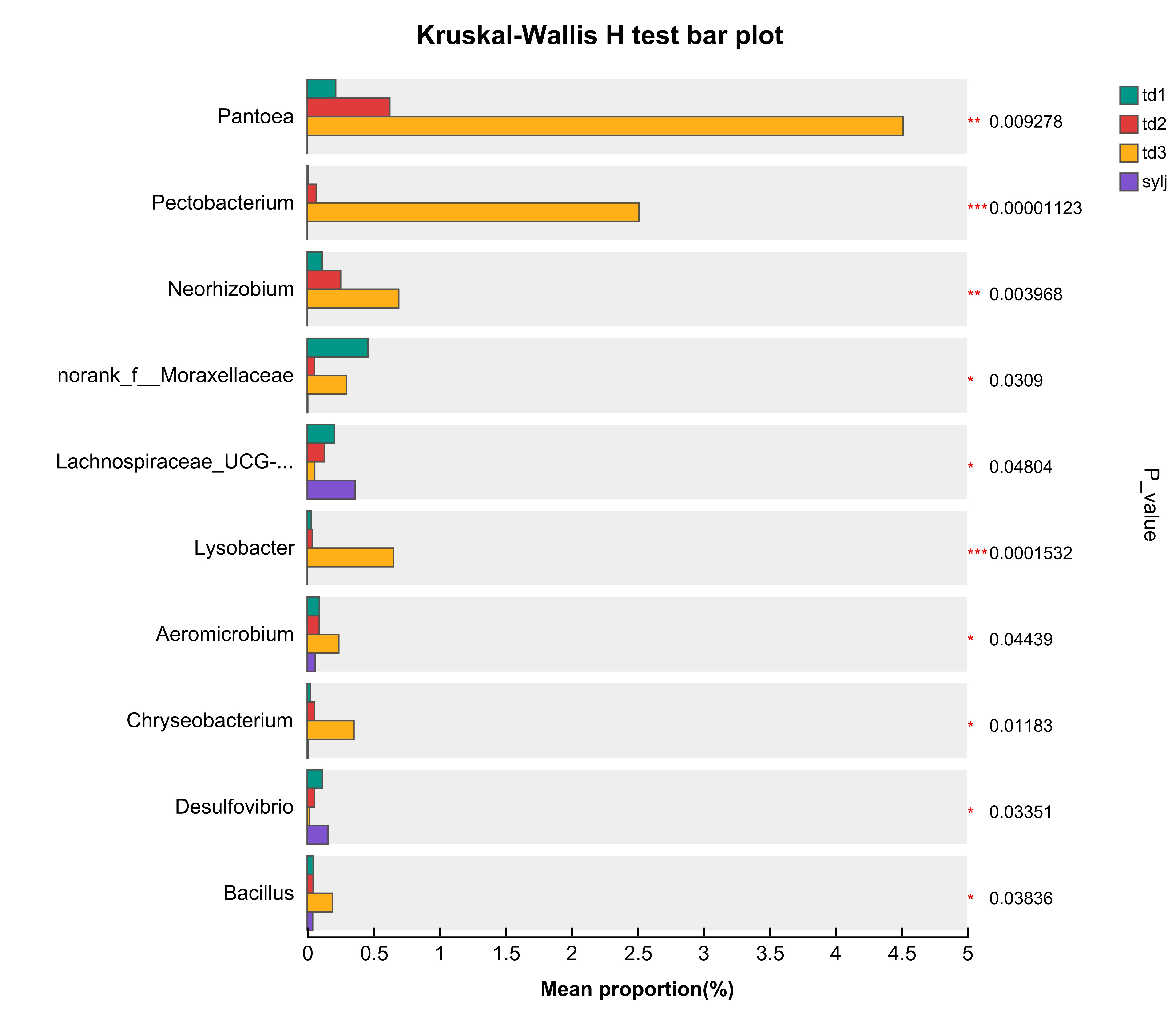

Supplement: Supplementary file 5 [file Image_5.JPEG]

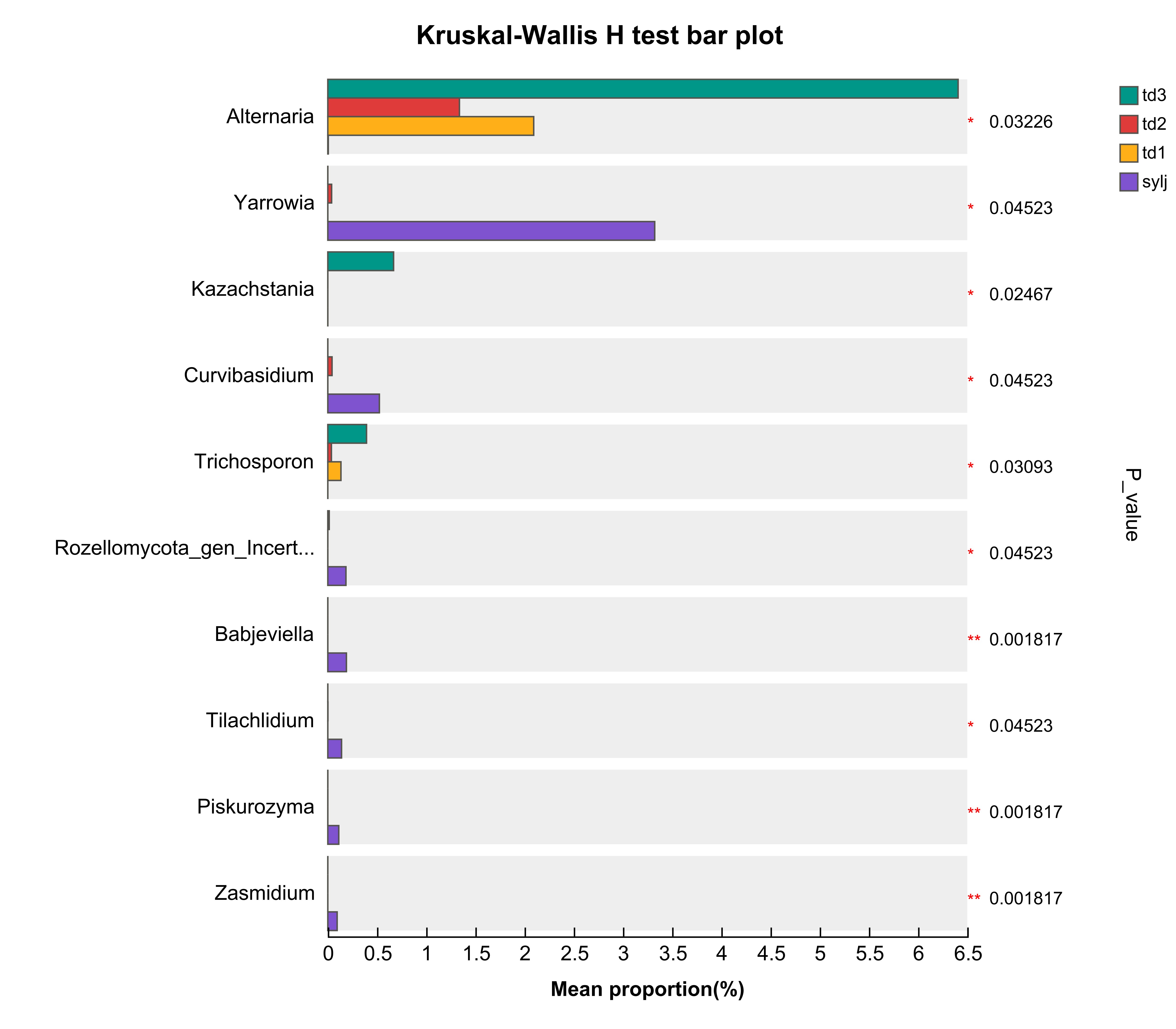

Supplement: Supplementary file 6 [file Image_6.JPEG]

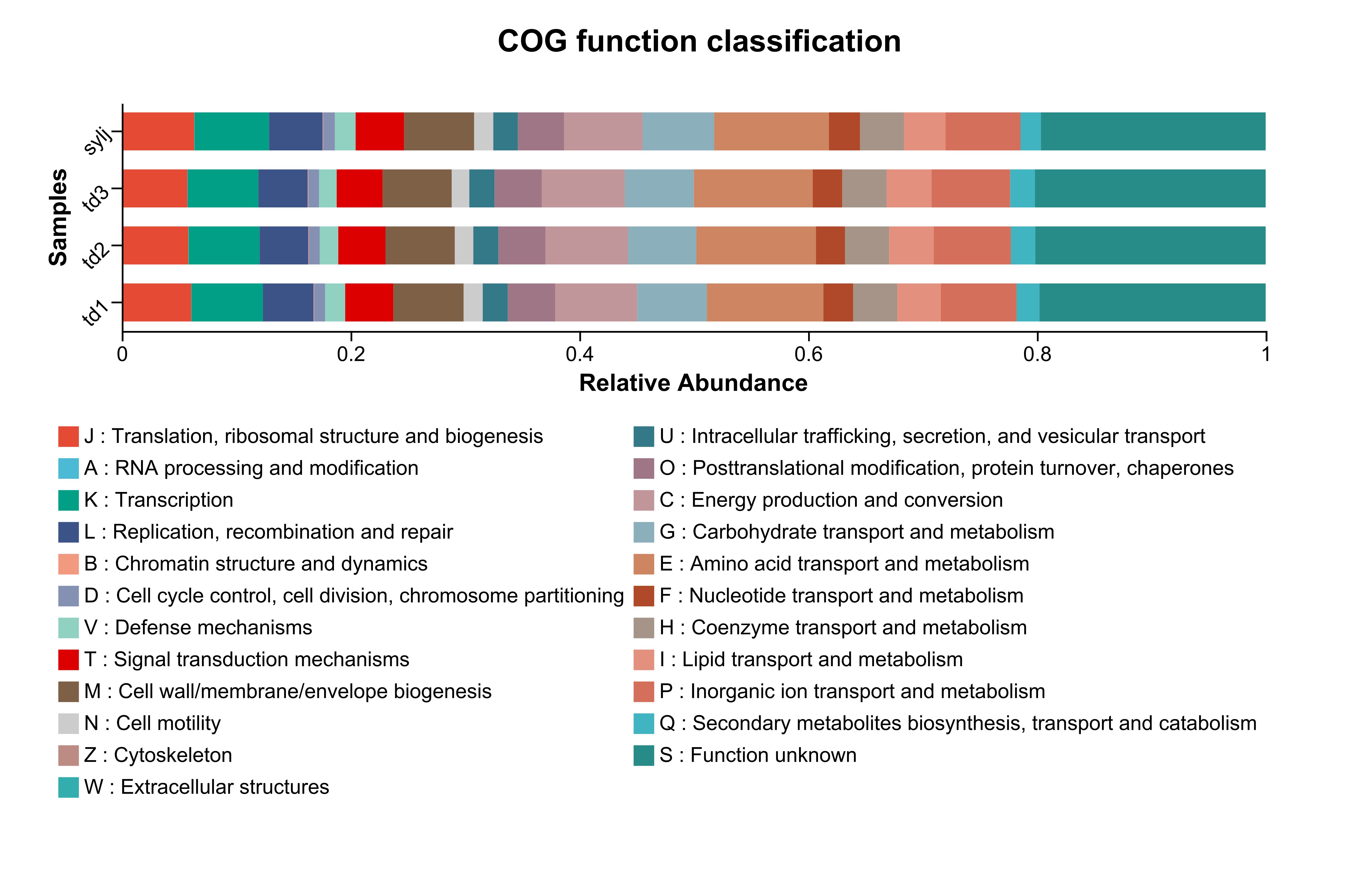

Supplement: Supplementary file 7 [file Image_7.JPEG]

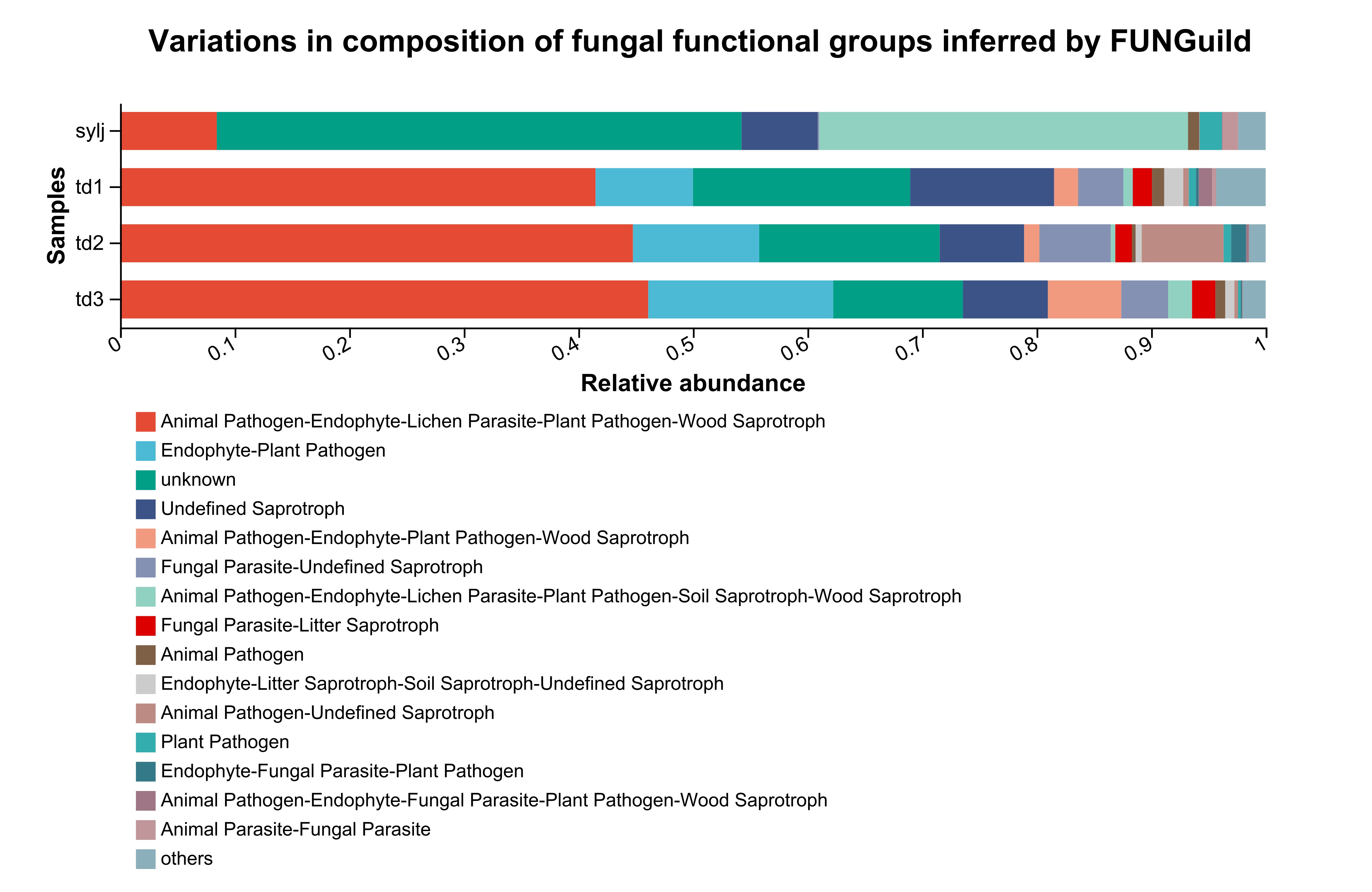

Supplement: Supplementary file 8 [file Image_8.JPEG]

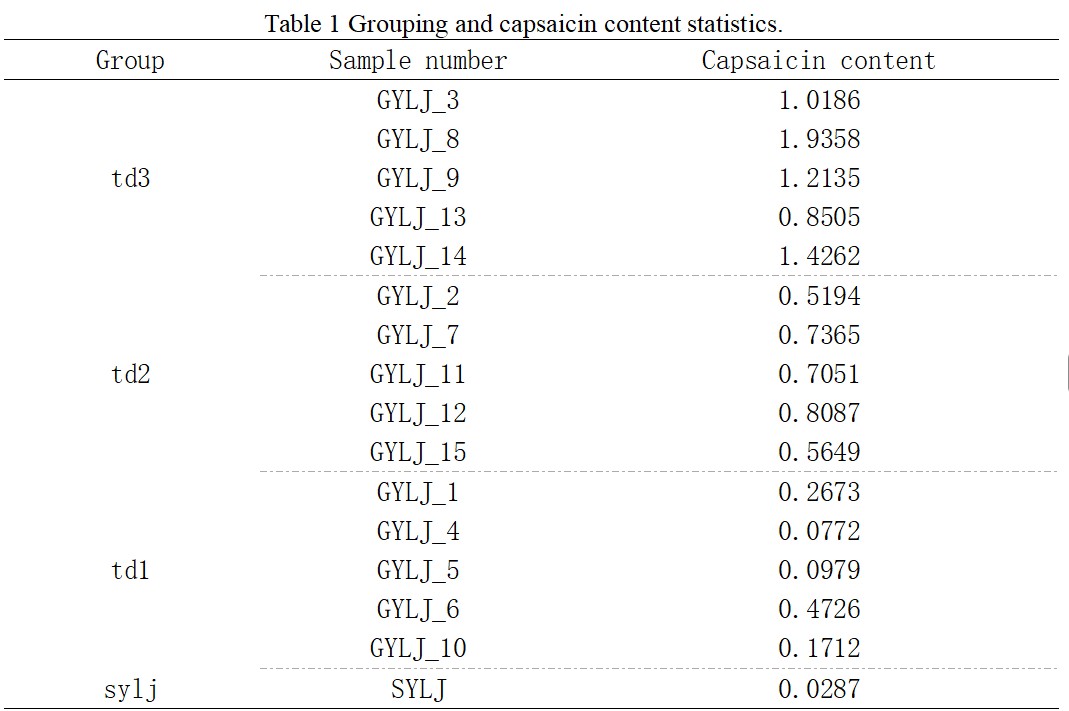

Supplement: Supplementary file 9 [file Image_9.JPEG]
